# Electroacupuncture versus Escitalopram for mild to moderate Post-Stroke Depression: A randomized non-inferiority trial

**DOI:** 10.3389/fpsyt.2024.1332107

**Published:** 2024-02-02

**Authors:** Feixiang Ma, Guiping Cao, Lu Lu, Yingling Zhu, Wanlang Li, Li Chen

**Affiliations:** ^1^ The First School of Clinical Medicine, Nanjing University of Chinese Medicine, Nanjing, Jiangsu, China; ^2^ Department of Rehabilitation, Yancheng Third People's Hospital, Yancheng, Jiangsu, China; ^3^ Department of Pharmacy, Yancheng Traditional Chinese Medicine (TMC) Hospital Affiliated to Nanjing University of Chinese Medicine, Yancheng, Jiangsu, China; ^4^ Department of Acupuncture, Liyang Hospital of Chinese Medicine, Changzhou, Jiangsu, China; ^5^ Department of Educational Services, Yancheng Third People's Hospital, Yancheng, Jiangsu, China; ^6^ Department of Acupuncture, Affiliated Hospital of Nanjing University of Chinese Medicine (Jiangsu Province Hospital of Chinese Medicine), Nanjing, Jiangsu, China

**Keywords:** electroacupuncture, Barthel Index, escitalopram, post-stroke depression, HAMD-17, inflammatory cytokine

## Abstract

**Objective:**

To explore the efficacy of electroacupuncture in treating post-stroke depression (PSD) by modulating the inflammatory response pathway.

**Methods:**

One hundred and fifty participants with mild or moderate PSD were randomly divided into 75 cases each in the electroacupuncture group (EA group) and escitalopram group (ESC group). In the EA group, 30 sessions of electroacupuncture were performed on the Baihui (GV 20), Yintang (GV 29), and the ipsilateral Taichong (LR 3) and Hegu (LI 4), simultaneous oral placebo for 40 days. The ESC group received oral escitalopram oxalate tablets 10mg to 20mg for 40 days, plus 30 sessions of sham electroacupuncture. The effectiveness of the treatment was evaluated by the Hamilton Depression Scale (HAMD-17), Self-Depression Scale (SDS), Modified Barthel Index Score (MBI), and the serum levels of IL-1β, IL-6, IL-10, TNF-α, and INF-γ.

**Results:**

There was no statistically significant difference in the baseline data, HAMD-17, SDS, MBI scores, and serum IL-1β, IL-6, IL-10, TNF-α, and INF-γ levels between the two groups of participants before the intervention (*P >*0.05). After treatment, HAMD-17 and SDS scores continued to decrease and MBI scores continued to increase in both groups. The differences were statistically significant at the 6th week and baseline, the 10th week and baseline, and the 10th week and the 6th week (all *P <*0.001). The differences in HAMD-17, SDS, and MBI scores between the two groups at the 6th week were not statistically significant (*P*=0.110, 0.115, 0.516, respectively); HAMD-17 scores and SDS scores in the EA group were lower than those in the ESC group at the 10th week, and the differences were statistically significant (*P*=0.002,0.026, respectively). In the 6th week, the serum levels of pro-inflammatory factors such as IL-1β, IL-6, TNF-α, and INF-γ were significantly lower in both groups compared with the baseline, while the level of anti-inflammatory factor IL-10 was significantly higher. The difference between the pre-and post-intervention intra-group comparisons was statistically significant (*P <*0.001), and the difference between the inter-group comparisons was not statistically significant (*P >*0.05). No serious adverse events occurred throughout the trial. Both therapies could safely and effectively improve HAMD-17, SDS, and MBI scores and modulate neuroinflammatory responses in PSD participants. After the treatment was stopped, some parameters were better in the EA group than the ESC group in a short time.

**Conclusion:**

Electroacupuncture is an effective, alternative to escitalopram for the treatment of mild-to-moderate PSD.

**Clinical trial registration:**

Chinese Clinical Trial Registry (ChiCTR2300072576).

## Introduction

Stroke is one of the most serious health issues worldwide, and has become the leading cause of death and adult disability in China (1). Post-stroke depression (PSD) is the most common neuropsychiatric disorder following stroke, with a prevalence rate ranging from 10% to 50% (2, 3). Age, income, gender, medical history, educational level, socioeconomic status, stroke subtype, and stroke severity were all identified as major risk factors for PSD (2, 4–6).

There is increasing evidence (7, 8) that the immune system plays an intricate role in the pathophysiological changes following cerebral ischemic injury. Subsequent cerebral neuroinflammation after ischemic brain injury promotes the infiltration of a large number of inflammatory cells (various subtypes of T cells, monocytes/macrophages, neutrophils, and a variety of inflammatory cells) into ischemia-affected areas, leading to brain cell death, which affects the patient’s neurological repair and poststroke complications. Many studies (9, 10) suggest cross-sectional correlations between inflammatory markers and differential severity of specific depression symptom dimensions. Many inflammatory cytokines, such as interleukin-1β (IL-1β), interleukin-6 (IL-6), interleukin-10 (IL-10), tumor necrosis factor-alpha (TNF-α), interferon-gamma(IFN-γ), etc. are thought to contribute to the evidence that inflammation plays an important role in PSD (11–16).

PSD was associated with increased functional disability, worse cognitive impairment, increased mortality, and an increased risk of stroke recurrence (17, 18). Therefore, timely and effective antidepressant treatment may result in improved functional outcomes. Currently, antidepressants and psychotherapy are the primary treatments for PSD. Selective 5-hydroxytryptamine reuptake inhibitors are a class of antidepressants widely used in clinical practice in China in recent years. These drugs selectively inhibit the recycling of 5-hydroxytryptamine in the presynaptic membrane, have little effect on norepinephrine, and almost do not affect the recycle of dopamine. It is characterized by high efficacy, few adverse effects, excellent tolerability, and convenient taking, mainly including fluoxetine, paroxetine, sertraline, fluvoxamine, citalopram, escitalopram, which is commonly known as the “Six Golden Flowers”. In particular, escitalopram is more popular due to its faster action (19) and better efficacy (20).

But in clinical practice, we found that some patients with PSD refused medication because they needed oral antihypertensive drugs, hypoglycemic drugs, lipid-lowering and plaque stabilizing drugs, and anti-platelet aggregation drugs at the same time, and they were worried about triggering or aggravating adverse reactions between drugs. Some patients also do not have enough knowledge about PSD, or they think that it is a shame to suffer from psychiatric disorders such as PSD, or they think that PSD is irrelevant and does not need treatment, or they think that psychotherapy is not cost-effective and thus refuse oral antidepressants or psychotherapy. Therefore, it seems crucial to develop an accessible alternative treatment strategy for patients with PSD.

Acupuncture is a therapy for diseases by inserting needles into specific acupuncture points of the body and has been used for thousands of years in China. Nowadays, electroacupuncture plays an important role in the clinical treatment of PSD because of its safety, efficacy, and lack of significant adverse effects (21, 22). The traditional theory of acupuncture poses a challenge in explaining its mechanism due to its high abstraction. Additionally, the potential mechanism of electroacupuncture for PSD remains unclear in modern medicine. This study aims to compare the efficacy of electroacupuncture and escitalopram in treating PSD by using the Hamilton Depression Scale (HAMD-17), Self-Depression Scale (SDS), Modified Barthel Index Score (MBI) and various inflammatory factors. The objective is to explore the relationship between the efficacy of electroacupuncture in PSD patients and inflammatory factors.

## Materials and methods

### Study design

We conducted a single-center parallel randomized controlled trial (RCT) with blinded participants and assessors. Throughout the trial, the assessors and data analyzer were unaware of the participant grouping and did not share information about the treatment received. The participants were uncertain whether they were taking a placebo medication or not. The acupuncturists, on the other hand, were aware of the participant grouping and provided verbal explanations or transient micro-stimulation of acupoints to address any doubts raised by the participants regarding the sham electroacupuncture treatment. This trial was approved by the Ethics Committee of Yancheng Third People’s Hospital on May 28, 2022 (Ethics-Audit-2022-13). Participants were inpatients or outpatients of the Department of Rehabilitation and Department of Neurology of the Yancheng Third People’s Hospital who were recruited to this trial between June 2023 and December 2023. This trial was registered at the China Clinical Trial Registration Center on June 16, 2023. Registration number ChiCTR2300072576.

### Diagnostic criteria

1. Refer to the diagnostic model of combining symptomatology and depression scale score recommended in the Chinese Expert Consensus on Clinical Practice of Post-stroke Depression.

2. Low mood as the main manifestation (self-expressed or observed), together with at least three of the following symptoms, lasting for more than one week.

①Loss of interest in daily activities, no sense of pleasure;

②Significant loss of energy and a persistent feeling of fatigue without cause;

③Psychomotor retardation or agitation;

④Low self-evaluation, or self-blame, or feelings of guilt, up to the level of delusion;

⑤Lack of decisiveness, difficulty in making associations, or a significant decrease in self-awareness of the ability to think;

⑥Insomnia, or early awakening, or excessive sleep;

⑦Recurrent thoughts of wanting to die, or suicidal attempts/behavior;

⑧Loss of appetite, or significant weight loss;

⑨Decreased libido.

3. The above symptoms occur within three months to one year after the stroke.

4. Exclude mental disorders caused by certain substances (e.g., medications, drugs, alcohol) or other somatic diseases (e.g., somatic diseases such as physical disability).

5. Rule out mental disorders caused by other major life events (e.g., bereavement).

6. HAMD-17 score ≥ 8.

### Inclusion criteria

1. Meet the above PSD diagnostic criteria; 2. Diagnosed with ischemic stroke by cranial CT or MRI; 3. Aged 20-70 years; 4. HAMD-17 scale score 8-24 (mild and moderate depression); 5. Stable condition, normal cognition, good communication and expression ability, and ability to cooperate in completing the assessment scales; 6. Voluntarily participate in the study and sign the informed consent form.

### Exclusion criteria

1. Women during pregnancy and lactation; 2. People who also suffer from other psychiatric diseases such as mania and schizophrenia; 3. People who cannot tolerate electroacupuncture treatment or people who are allergic to drugs; 4. People who have participated in other clinical trial studies in the last 3 months; 5. People who have suicidal and self-harming tendencies; 6. People who have received other antidepressant treatment methods within three months;7. Contraindications to acupuncture such as localized infection and skin breakage at acupoints.

### Sample size calculation

According to the pre-experiment, it was known that the HAMD-17 scores were 14.8 ± 3.4 and 13.1 ± 3.1 in the EA group and the ESC group after the intervention, respectively. Considering a 5% false positive error (α =0.05, two-sided) and 85% power (β =0.15) during the experiment. 67 patients per group were measured to be required by the G-power(version 3.1)sample size calculation software, and combined with a shedding rate of about 10%, it was planned to include at least 75 patients per group.

### Randomization

A researcher who was not involved in the trial conducted the randomization process. SPSS 26.0 (SPSS Inc., Chicago, IL, USA) was utilized to generate 150 non-repeating ordered random numbers. These numbers were then subjected to “visual sorting” at a 1:1 ratio, and the relationship between the random numbers and the groups was recorded. Subsequently, random numbers were placed into opaque envelopes and the participants were randomized to select one envelope in the order of their visit to the clinic. According to the previous relationship schedule, the groupers divided the participants into an EA group and an ESC group of 75 each.

### Intervention

#### Basic treatment

All participants who met the inclusion criteria received secondary stroke prevention medications, including antihypertensive, hypoglycemic, antiplatelet aggregation, lipid-lowering, and plaque stabilization medications, as well as systematic rehabilitation treatments such as exercise therapy, neuroelectric stimulation, and hand function training, based on their individual needs. However, acupuncture therapy was not administered.

#### Electroacupuncture group

Point selection: Yintang (GV 29), Baihui (GV 20), bilateral Taichong (LR 3), bilateral Hegu (LI 4).

**Figure 1:**
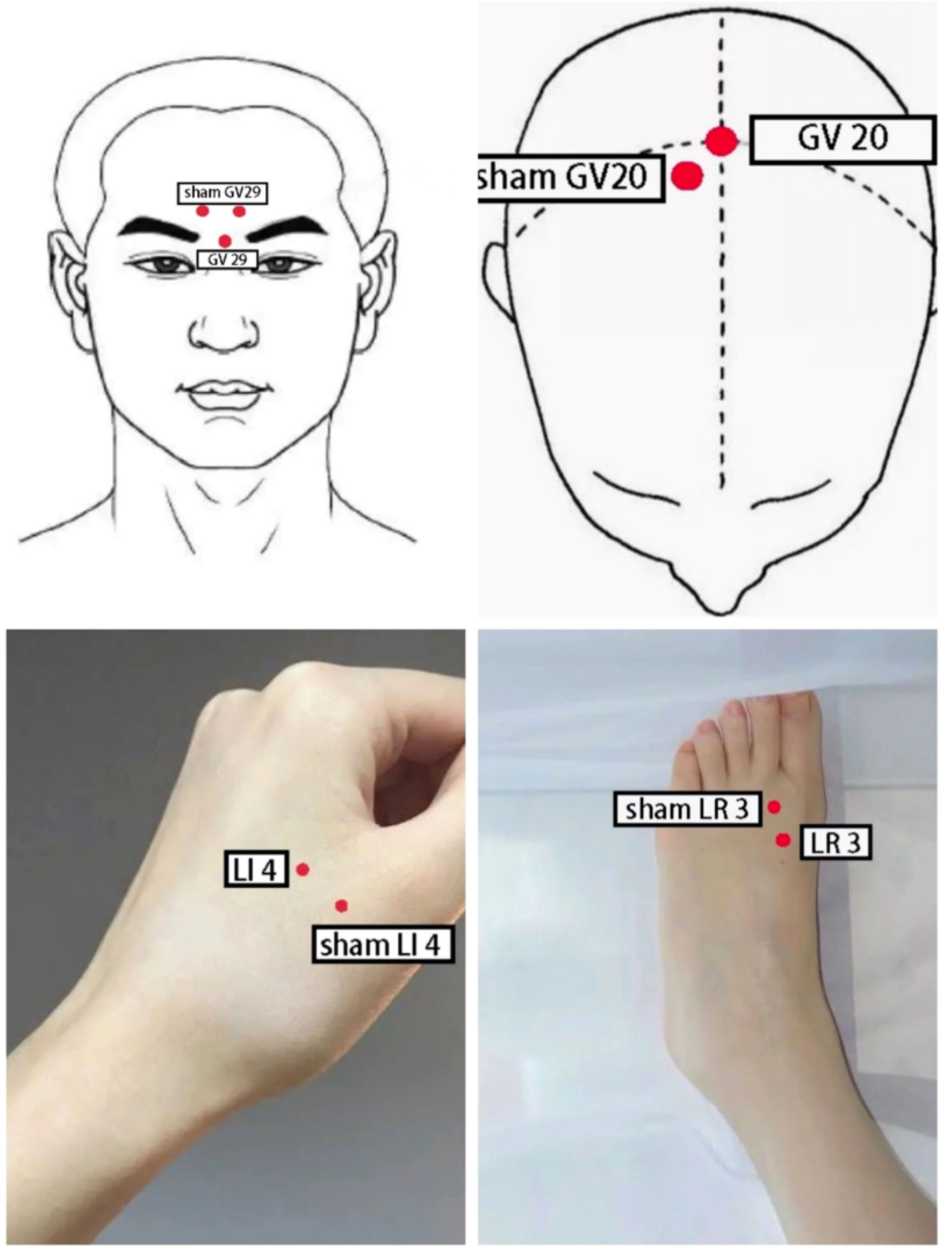
Schematic diagram of acupoint localization for electroacupuncture and sham electroacupuncture.

Operation: The patient was positioned supine and the local skin was disinfected with iodophor. A sterile acupuncture needle measuring 0.30mm*25mm (Suzhou Dongbang Medical Instrument Co., Ltd.) was selected and inserted into Baihui (GV 20) at a 45° angle to the scalp, approximately 15-20mm deep. The Yintang (GV 29) was punctured downward at a 15° angle, approximately 10-15mm deep. Lateral Taichong (LR 3) and Hegu (LI 4) were pierced perpendicularly to the body surface using sterile acupuncture needles measuring 0.30mm*40mm, approximately 25-30mm deep. By twirling, lifting, or thrusting the acupuncture needle, the participants were getting Deqi (they feel soreness, heaviness, numbness, distention, or pain in the local area of the acupoints). Then, Baihui (GV 20) and Yintang (GV 29), the ipsilateral Taichong (LR 3) and Hegu (LI 4) were connected to the Indy KWD-808 I-type pulse electronic acupuncture therapeutic apparatus. The treatment frequency of 2/15 Hz was selected, and the intensity was based on the tolerance of the participants. Once a day, 30 minutes each time, 5 times a week, a total of 30 sessions for 6 weeks. As shown in **Figure 1**.

The acupuncturist was performed by a registered acupuncture doctor with more than 10 years of work experience.

During the same period, nurses gave every participant placebo tablets to take orally per day before breakfast for 40 days. Depending on the severity of depression, they can take two tablets daily. The Pharmacy Department of Yancheng Hospital of Traditional Chinese Medicine produces Escitalopram Oxalate Tablets according to their appearance, with dextrin, soluble starch, lactose, maltodextrin and other raw materials as the main raw materials. The shape and packaging are consistent with the original drug.

#### Escitalopram group

Nurses distributed escitalopram oxalate tablets (Baisaile, Registration NO. H20080599, Jingwei Pharmaceutical Co., Ltd.), and participants took them orally before breakfast. Depending on the severity of depression, the dose is 10-20 mg daily for 40 days. In addition to medication, participants also received sham electroacupuncture. They were placed in the supine position, and the 0.5-1 cm area near Baihui (GV 20), Yintang (GV 29), Taichong (LR 3), and Hegu (LI 4) were selected as acupoints. After routine disinfection of the local skin with iodophor, 0.30mm×15mm sterile disposable needles (Suzhou Dongbang Medical Instrument Co., Ltd.) were used to pierce into the skin at an angle of 15° and a depth of 3 to 5 mm to produce a sense of pain to the participants. Then, three groups of electroacupuncture in accordance with the method of the EA group were connected, but the frequency and intensity were zero. The treatment was performed once a day for 30 minutes, five times a week, a total of 30 sessions in six weeks.

Both groups of participants were given a harmonized and standardized psychotherapy by a psychotherapist for 5 days after the termination of intervention. During the treatment, a designated investigator was responsible for documenting adverse events experienced by participants and making appropriate decisions to terminate the trial or not.

### Primary outcomes

**Hamilton Depression Scale (HAMD-17):** A British psychiatrist, Hamilton, developed it in 1960. The scale consists of 17 entries scored from 0 to 4 according to their severity. Two researchers who were not involved in the grouping completed the assessment through questioning and observation within 20 minutes, and an average score was determined as the final score. Higher scores indicate more severe depression.

### Secondary outcomes

**Zung Self-rating Depression Scale (SDS):** It was created in 1965 by Duke University psychiatrist Dr. William WK Zung. The scale consists of 20 items, each rated from 0 to 4. Participants filled in the scale individually within 10 minutes according to their actual situation. The total score was multiplied by 1.25 to get the final score.

**Modified Basel Index (MBI):** It was modified from the Barthel Index by Shah et al. in 1989. The scale includes 10 items with each scoring from 0 to 15. Two investigators who were not involved in the grouping completed the assessment within 20 minutes by questioning and observing, and the average score was used as the final score.

Assessors evaluated the participants with the scale at baseline, within 24 hours when the intervention was completed at 6th week, and at the follow-up at 10th week.

### Inflammatory cytokine content test

Participants had their blood withdrawn from the elbow vein in the morning by a heparin-containing anticoagulant tube in a fasting state for approximately 5 mL, and the blood was sent to the laboratory center within 2 hours. The serum IL-1β, IL-6, IL-10, TNF-α, and INF-γ levels of the participants were measured by immunofluorescence method with the BD-FACSCanto-II flow cytometer (BD Rhapsody, USA) at baseline and within 48 hours after the end of the 6-week intervention. If the participants had other infectious diseases, such as respiratory infections within 24 hours before sampling, the research team would evaluate and decide whether the case should be eliminated or the time for sampling, then record the relevant information and arrange the review time.

### Statistical analysis

SPSS 26.0 statistical software (SPSSInc., Chicago, IL., USA) was used for data description and statistics. Enumeration data were analyzed using the chi-square test, the measurement data that obeyed the normal distribution were expressed as the mean ± standard deviation (mean ± SD). The paired-samples t-test was used for intra-group comparison, and the two-independent-samples t-test for inter-group comparison. Measurement data that do not conform to the normal distribution were expressed as the median and four-digit range. The Wilcoxon signed-rank test was used for intra-group comparisons, and the Mann-Whitney U test was used for intra-group comparisons. All differences were considered statistically significant at *P <*0.05.

## Results

### General information

**Figure 2:**
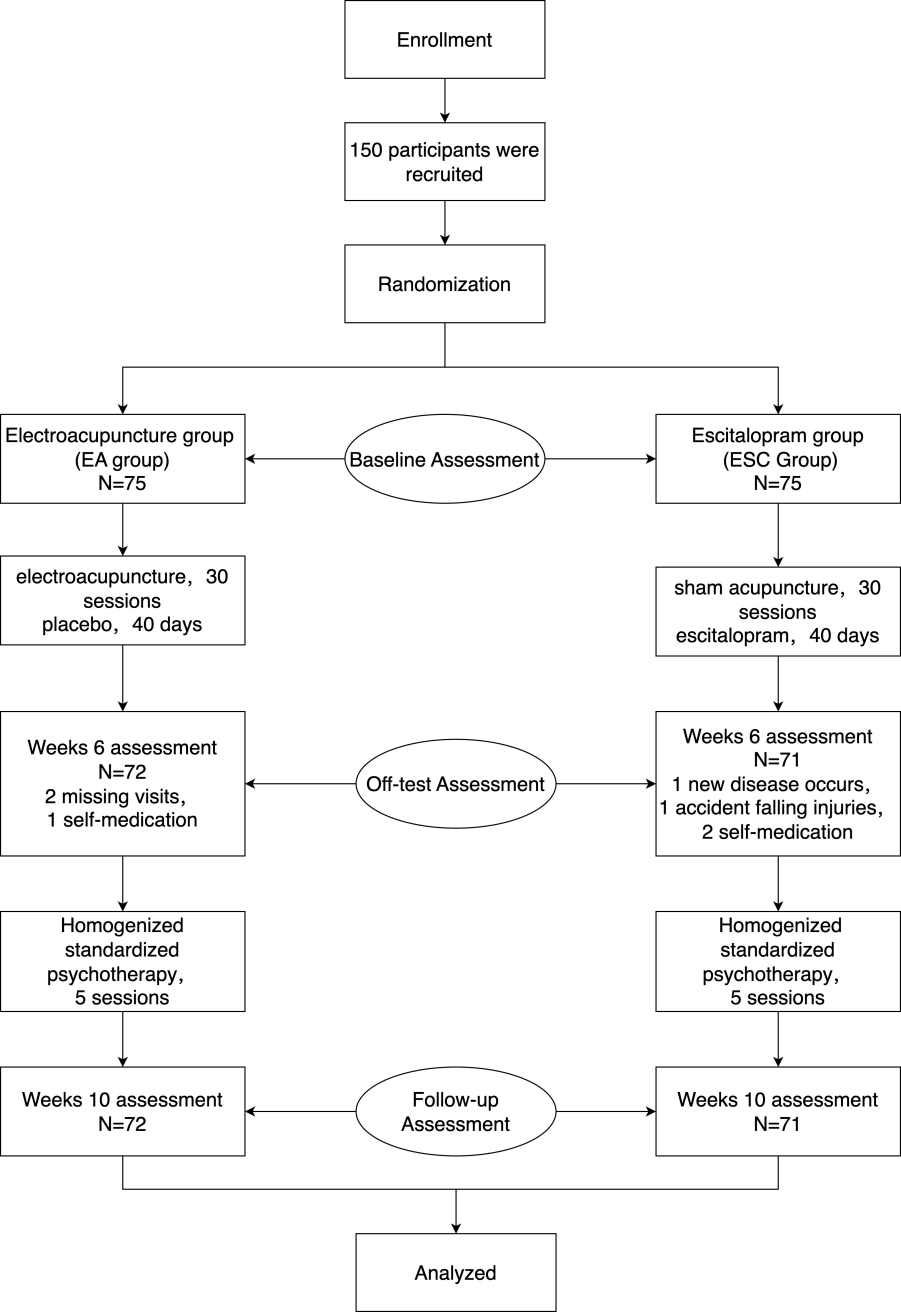
Trial flowchart.

During the study, two cases in the EA group were discharged due to missing visit, one due to self-administration of other antidepressants, and one in the ESC group was discharged due to the participant’s complication with other diseases, two cases continued oral escitalopram tablets on their own after complication with withdrawal syndrome, and one case was discharged due to an unintentional fall. 72 cases in the EA group and 71 cases in the ESC group finally completed this study. There was no statistically significant difference between the two groups of participants in gender, age, disease duration, and disease severity (including HAMD score) (*P*>0.05), and the baseline data were comparable. The trial flow chart is shown in **Figure 2**. Case details are shown in **Table 1**.

**Table 1:** Clinical characteristics of two groups of patients.

Variable	EA Group(N=72)	ESC Group(N=71)	*P* value
Age (years, range)	54.65 ± 7.07(52.99,56.31)	56.10 ± 5.93(54.70,57.50)	0.107
Gender (male/female)	42/30(58.3%)	44/27(62.0%)	0.657
Hypertension (yes/no)	35/37(48.6%)	42/29(59.2%)	0.206
Hyperlipidemia (yes/no)	45/27(62.5%)	47/24(66.2%)	0.644
Diabetes (yes/no)	37/35(51.4%)	39/32(54.9%)	0.671
Stroke Course (weeks, range)	29.75 ± 8.84 (27.67,31.83)	29.06 ± 8.20 (27.12,31.00)	0.512

### HAMD-17 scores

**Figure 3:**
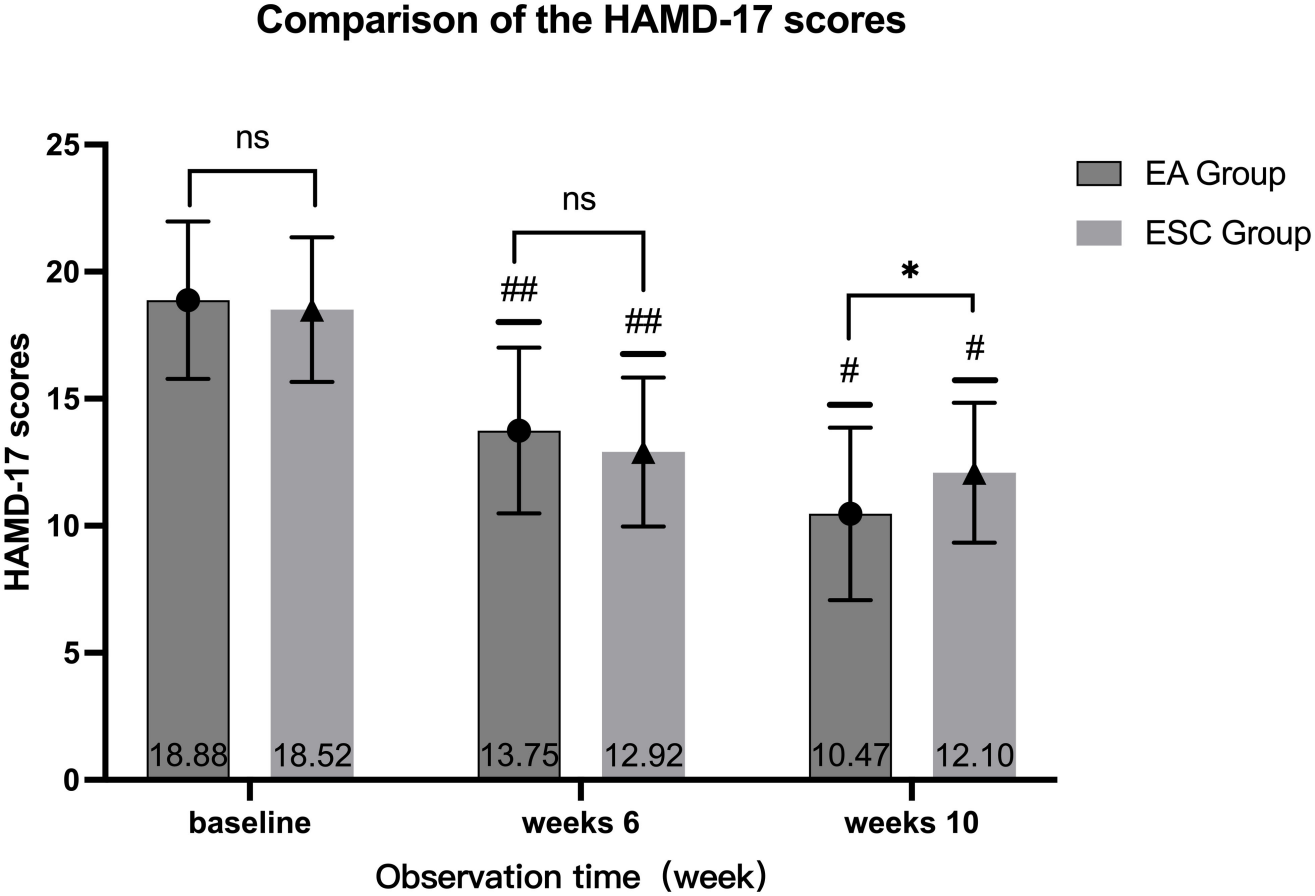
Comparison of different times within the group, 6th week VS baseline in each group, ^##^ *P <*0.001; 10th week VS baseline, 10th week VS 6th week in each group, ^#^ *P <*0.001; Compared with ESC group in the same time, ns *P >*0.05, ^*^ *P <*0.05.

After 40 days of different interventions, the HAMD-17 scores in the EA group decreased from baseline (18.88 ± 3.10) to 13.75 ± 3.26 (28% decrease rate) at the 6th week and 10.47 ± 3.39 (45% decrease rate) at the 10th week, while in the ESC group, they decreased from 18.52 ± 2.85 to 12.92 ± 2.93 (31% decrease rate), and 12.10 ± 2.75 (35% decrease rate), respectively. Scores in each group at the 6th week and 10th were lower than the baseline, and at the 10th week were lower than the 6th. The differences were statistically significant (*P <*0.001). There were no significant differences in HAMD-17 scores between the two groups at baseline (*P* =0.479) and 6th week (*P* =0.110). In the 10th week, the EA group was lower than the ESC group, and the difference was statistically significant (*P* =0.002). As shown in **Figure 3**.

### SDS scores

**Figure 4:**
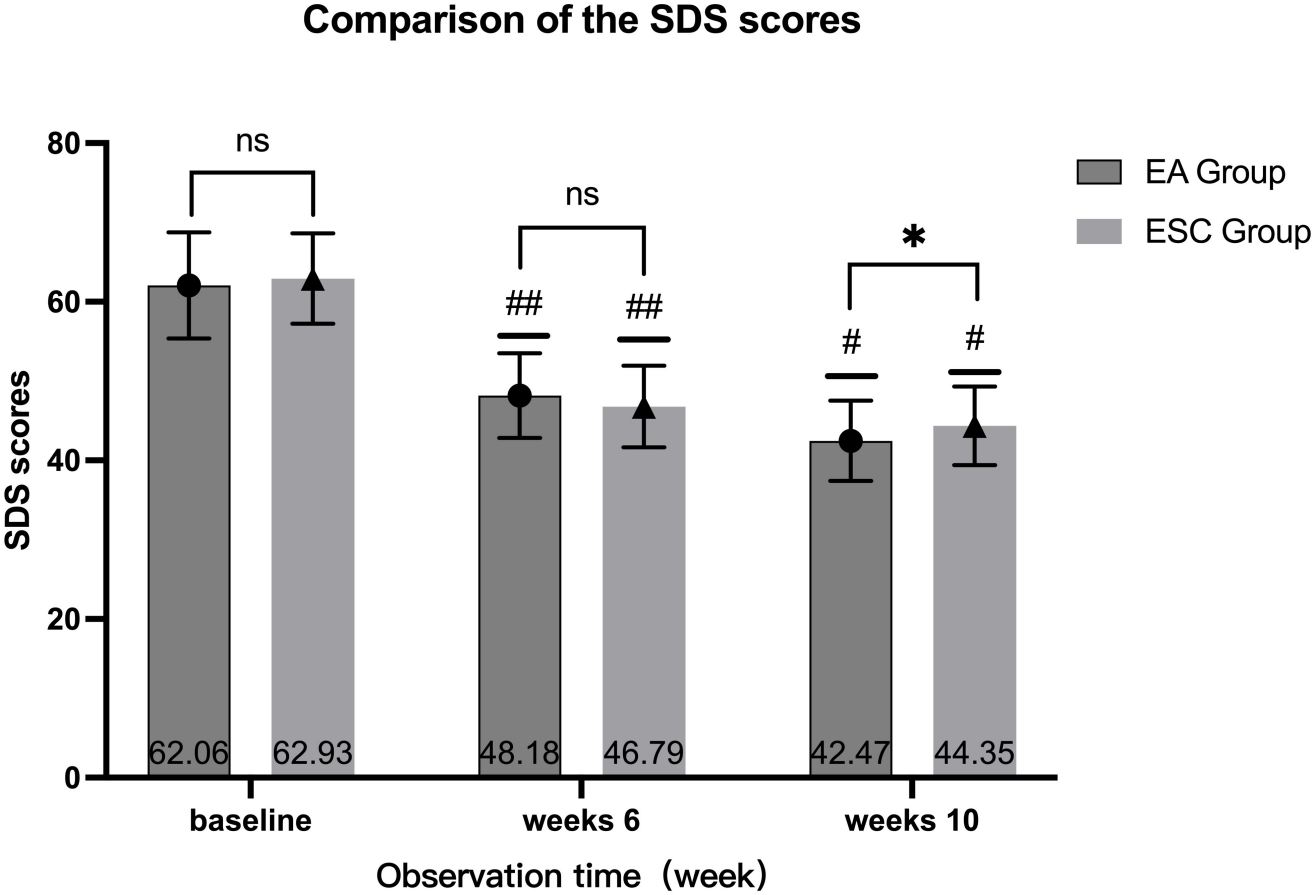
Comparison of different times within the group, 6th week VS baseline in each group, ^##^ *P <*0.001; 10th week VS baseline, 10th week VS 6th week in each group, ^#^ *P <*0.001; Compared with ESC group in the same time, ns *P >*0.05, ^*^ *P <*0.05.

After 40 days of different interventions, the SDS scores of the EA group were 48.18 ± 5.33 and 42.47 ± 5.07 in 6th week and 10th, respectively, while the ESC group were 46.79 ± 5.15 and 44.35 ± 4.95. Scores at the 6th week and 10th were lower than the baseline (62.06 ± 6.69,62.93 ± 5.71, respectively), and at the 10th week were lower than the 6th. The differences were statistically significant (*P <*0.001). There were no significant differences in SDS scores between the two groups at baseline (*P* =0.402) and 6th week (*P* =0.115). In the 10th week, the EA group was lower than the ESC group, and the difference was statistically significant (*P* =0.026). As shown in **Figure 4**.

### MBI scores

**Figure 5:**
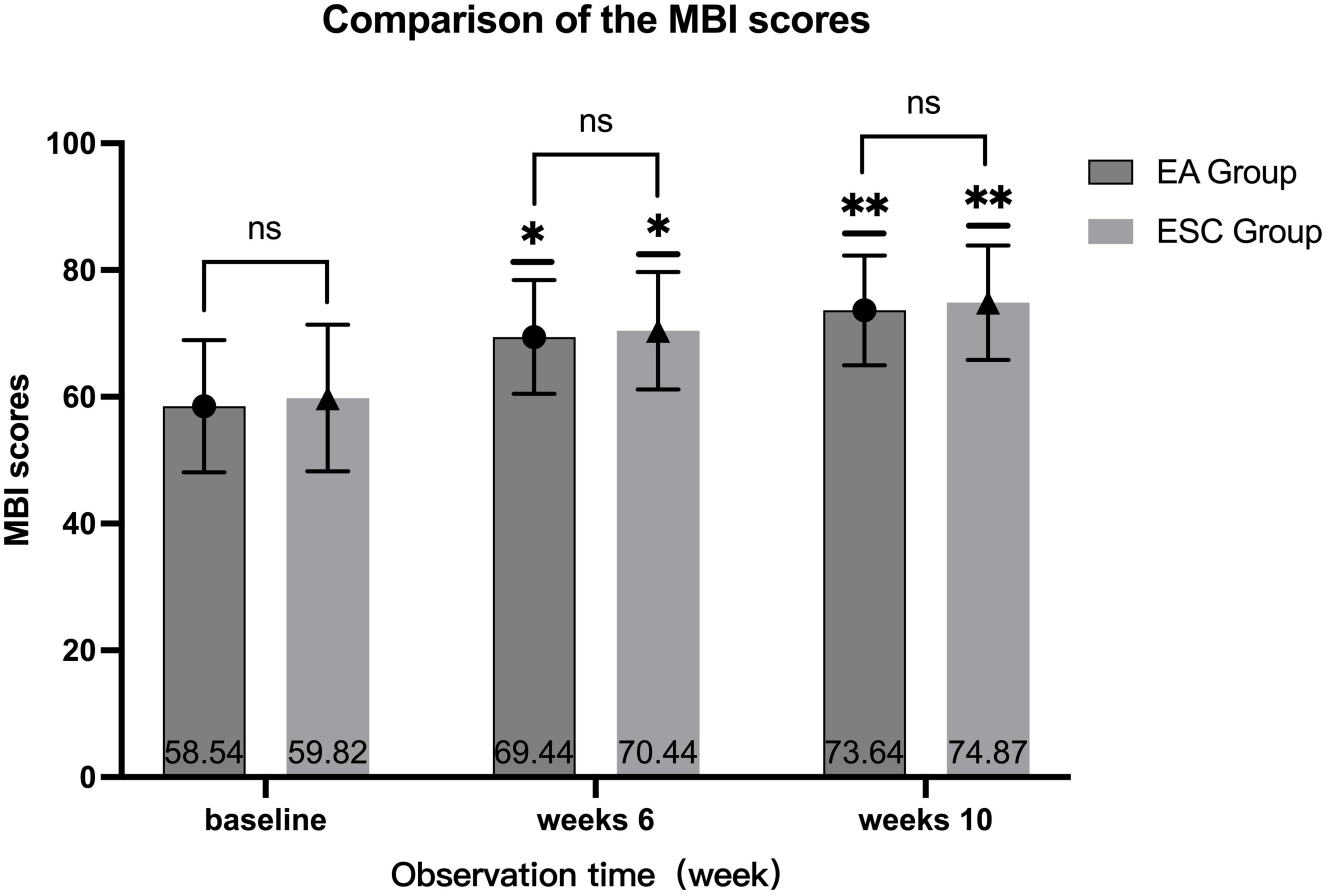
Comparison of different periods within the group, 6th week VS baseline in each group, ^*^ *P*<0.001, 10th week VS baseline, 10th week VS 6th week in each group, ^**^ *P*<0.001; Compared with ESC group in the same period, ns *P >*0.05.

After 40 days of different interventions, the MBI scores of the EA group were 69.44 ± 8.97 and 73.64 ± 8.64 in 6th week and 10th, respectively, while the ESC group were 70.44 ± 9.26 and 74.87 ± 9.03. Scores at the 6th week and 10th were higher than the baseline (58.54 ± 10.42,59.82 ± 11.59, respectively), and at the 10th week were higher than the 6th. The differences were statistically significant (*P <*0.001). There was no significant difference in the MBI score between the two groups in the baseline (*P* =0.490), 6th week (*P* =0.516) and 10th week (*P* =0.405). As shown in **Figure 5**.

### Inflammatory factors

After 40 days of different interventions, the levels of IL-1β, IL-6, TNF-α and INF-γ in each group were lower than the baseline levels in the 6th week, while the levels of IL-10 were higher. When compared within the group, the difference was statistically significant (*P <*0.05). At the same time, there was no statistical difference in the levels of various inflammatory factors between the groups (*P <*0.05).

The comparison of the outcomes before and after intervention between the two groups is detailed in **Table 2**.

**Table 2:** Comparison of outcomes between the two groups before and after intervention.

Outcome	EA Group(N=72)	ESC Group(N=71)	Between-group differences (95% CI)	*P* value
HAMD-17				
Baseline	18.88 ± 3.10(18.15,19.60)	18.52 ± 2.85(17.85,19.20)	0.63-1.34	0.479
6th week	13.75 ± 3.26^⋆^(12.98,14.52)	12.92 ± 2.93^⋆^(12.22,13.61)	0.19-1.86	0.110
10th week	10.47 ± 3.39^⋆▲^(9.67,11.27)	12.10 ± 2.75^⋆▲▪^(11.45,12.75)	0.60-2.65	0.002
SDS				
Baseline	62.06 ± 6.69(60.48,63.63)	62.93 ± 5.71(61.58,64.28)	1.18-2.93	0.402
6th week	48.18 ± 5.33^⋆^(46.93,49.43)	46.79 ± 5.15^⋆^(45.57,48.01)	0.34-3.13	0.115
10th week	42.47 ± 5.07^⋆▲^(41.28,43.66)	44.35 ± 4.95^⋆▲▪^(43.18,45.52)	0.22-3.54	0.026
MBI				
Baseline	58.54 ± 10.42(56.09,60.99)	59.82 ± 11.59(57.07,62.56)	2.37-4.92	0.490
6th week	69.44 ± 8.97^⋆^(67.34,71.55)	70.44 ± 9.26^⋆^(68.25,72.63)	2.02-4.00	0.516
10th week	73.64 ± 8.64^⋆▲^(71.61,75.67)	74.87 ± 9.03^⋆▲^(72.74,77.01)	1.69-4.16	0.405
IL-1β(pg/ml)				
Baseline	10.59 ± 3.67(9.72,11.45)	10.99 ± 3.41(10.19,11.80)	0.76-1.58	0.492
6th week	5.62 ± 2.57^⋆^(5.02,6.23)	6.14 ± 2.71^⋆^(5.50,6.78)	0.35-1.39	0.241
IL-6(pg/ml)				
Baseline	36.21 ± 15.54(32.56,39.86)	36.57 ± 14.86(33.05,40.09)	4.67-5.39	0.887
6th week	23.16 ± 13.03^⋆^(20.10,26.22)	22.13 ± 9.46^⋆^(19.89,24.37)	2.74-4.80	0.589
IL-10(pg/ml)				
Baseline	10.12 ± 4.41(9.08,11.16)	9.49 ± 4.59(8.40,10.57)	0.86-2.12	0.402
6th week	17.22 ± 4.45^⋆^(16.17,18.26)	16.75 ± 3.99^⋆^(15.81,17.70)	0.93-1.86	0.512
TNF-α(pg/ml)				
Baseline	12.94 ± 3.35(12.16,13.73)	12.93 ± 3.17(12.18,13.68)	1.07-1.09	0.984
6th week	7.37 ± 3.48^⋆^(6.56,8.19)	7.49 ± 3.29^⋆^(6.71,8.26)	1.00-1.24	0.839
INF-γ(pg/ml)				
Baseline	15.52 ± 3.86(14.62,16.43)	16.06 ± 3.33(15.27,16.85)	0.66-1.73	0.377
6th week	10.11 ± 3.90^⋆^(9.19,11.02)	10.58 ± 3.37^⋆^(9.78,11.37)	0.73-1.68	0.440

Within-group comparisons with the baseline, ^⋆^ *P* <0.001, within-group comparisons with the 6th week, ^▲^ *P* <0.001; between-group comparisons with the EA group at the same time, ^▪^ *P* <0.05.

Four participants in the EA group experienced local hematoma and pain during the study, three participants in the ESC group experienced transient dizziness, and two others experienced mild drowsiness. Five participants experienced chest tightness, irritability, insomnia, anxiety, and other withdrawal symptoms that lasted less than 48 hours. All participants recovered without any special care or treatment. This indicates that electroacupuncture and escitalopram are both safe treatments for post-stroke depression.

## Discussion

In this study, we discovered that both electroacupuncture and escitalopram could be effective in reducing the participants’ HAMD-17 and SDS scores, as well as their serum levels of pro-inflammatory factors such as IL-1β, IL-6, TNF-α, and INF-γ, while elevating MBI scores and serum levels of anti-inflammatory factors like IL-10 after 40 days of various interventions. In intra-group comparisons, each group showed superior results to the baseline for all outcomes at the 6th and 10th week, with the 10th week better than the 6th.There was no statistically significant difference in each outcome between the two groups in the 6th week. In the 10th week, the HAMD-17 and SDS scores of the EA group were lower than the ESC group (mean deviation 1.63 ± 0.52, 95% CI 0.60-2.65, and 1.88 ± 0.84, 0.22-3.54, respectively), and the difference between groups was statistically significant.

It suggested that the effectiveness of electroacupuncture and escitalopram was similar in improving the depressive state in patients with mild to moderate depression after ischemic stroke by regulating the inflammatory response. Moreover, electroacupuncture has demonstrated superior short-term sustainability compared to escitalopram. No major adverse events occurred throughout the study, and the results suggested that electroacupuncture may be a safe and effective therapy (23) for mild to moderate PSD, albeit with our small sample size.

Although the medical community had recognized PSD more than 100 years earlier (24), it was until 1977 that the concept of PSD was first proposed by Folstein (25). During these 50 years of systematic research, scientists have not yet reached a consensus on the pathogenesis of PSD (26, 27). PSD did not have a clear name in ancient Chinese medical literature. The acupuncture principles of PSD generally refer to the treatment principles of depression disease, and depressive syndrome. Traditional acupuncture theory considers that PSD is caused by DU-Yang deficiency, Qi stagnation, and blood stasis.

The Baihui (GV 20) and Yintang (GV 29) belong to the Governor’s Vessel, which can regulate the spirit and invigorate Yang energy; the Taichong (LR 3) and Hegu (LI 4) (commonly known as the “four guan points “) belong to the Liver and Large Intestine meridians, respectively, with the former ascending Qi and the latter descending Qi. The combination of these points can acquire the effects of dispersing stagnated hepatoqi and coordinating Qi and blood, making them the main acupoints to be chosen for the therapy of PSD. Studies by Yin (28), Lu (29), and Li (22) have also demonstrated the effectiveness of these acupoints in treating PSD, which is generally consistent with our findings.

However, many studies (28, 30, 31) are presently integrating acupuncture with other antidepressant treatments, such as pharmacotherapy, making it difficult to assess the efficacy of each method and making it unsuitable for patients who require a single therapy. Therefore, this study compared electroacupuncture with escitalopram, which is commonly used in clinical practice for its efficacy, and added placebo and sham electroacupuncture to demonstrate the independent therapeutic effect of electroacupuncture, find the main efficacy factors in the combined therapy, and avoid duplication of treatment in patients as much as possible. The effect of electroacupuncture and escitalopram was comparable in terms of HAMD-17, SDS scores, etc., and the sustainability in the short-term effect of electroacupuncture was superior to escitalopram, indicating that electroacupuncture can be used alone in treating PSD. Additionally, electroacupuncture has also demonstrated fewer side effects and a lower risk of dependency compared to escitalopram, this makes it a more advantageous option for individuals seeking alternative therapies.

Although some studies (9) have argued that PSD is not associated with inflammatory responses, more and more studies (11, 32) have shown that inflammatory responses are closely associated with PSD, especially pro-inflammatory factors such as IL-1β, IL-6, TNF-α, INF-γ, and the inflammation suppressor IL-10. Previous studies (16, 33) have shown that the immune system, as a diffuse sensory organ of the brain, can interact with the immune system, a diffuse sensory organ of the brain that can form a bidirectional contact network with the brain. After the stroke, human immune cells are activated, releasing various inflammatory cytokines that infiltrate and activate microglia in the brain, leading to neuronal necrosis and the production of reactive oxygen radicals, leading to changes in neurotransmitters in the brain through an inflammatory cascade, resulting in emotional, cognitive and behavioral changes, and ultimately depression.

Electroacupuncture has been used to treat PSD for years but its mechanism is not fully elucidated (23, 34, 35). The current dominant mechanisms of effect include regulating monoamine neurotransmitters and receptors (36) such as 5-hydroxytryptamine (5-HT) (31, 37), norepinephrine (NE) (38) and dopamine (DA) (39); modulating amino acid neurotransmitters such as glutamate (40); inhibiting the hypothalamic-pituitary-adrenal axis (HPA) (41) to improve neuroendocrine dysfunction; enhancing the expression of brain-derived neurotrophic factor (BDNF) (42)to inhibit hippocampal neurons apoptosis (43); reducing oxidative stress and neuroapoptosis (44); changing intestinal microbiota (45), regulating mitochondrial homeostasis (46), etc.

The anti-inflammatory pathway is one of the hot spots in clinical research. Song (47) found that both EA and fluoxetine had an anti-inflammatory effect by reducing IL-1β in major depressive disorder patients. Zhao (48) found that acupuncture can drive anti-inflammatory effects by Meta-analysis. Li (49, 50)found acupuncture plays a positive role in anti-depression in rats due to its effects in inhibiting the expression of NLRP3 inflammasome activation and reducing proinflammatory cytokines in the microglia and the prefrontal cortex. Dong (51) suggested that the improvement in depressive-like behaviors induced by EA is likely achieved via activation of the tissue plasminogen activator (tPA)/brain-derived neurotrophic factor (BDNF)/tyrosine kinase receptor B (TrκB) signaling pathway in the prefrontal cortex. Coincidentally, it has also been shown (9, 52–55) that escitalopram can treat PSD by modulating the inflammatory response, and our study validates this conclusion. However, the mechanism is not yet clarified. The general opinion (56) is that it affects the HPA axis (57, 58) and has an anti-inflammatory effect on depression by modulating synaptic excitability in the hippocampus (59).

Combined with previous research results, for the purpose of this study, we suggest that electroacupuncture can inhibit the overactivity of the hypothalamic-pituitary-adrenal axis (41), down-regulate microglia activation (60) in the prefrontal cortex (49) and hippocampus (61, 62), reduce inflammatory responses (63) in the brain at stroke sites, repair brain tissue in damaged areas (64), and negatively feedback regulate the release of pro-inflammatory factors such as IL-1β, IL-6, TNF-α, and The negative feedback regulates the release of pro-inflammatory factors such as IL-1β, IL-6, TNF-α, INF-γ and activates the secretion of anti-inflammatory factor IL-10, which reduces inflammatory damage in the brain by regulating the synthesis and secretion of inflammatory mediators and balancing the expression of inflammatory factors, thereby improving the symptoms of PSD patients. This mechanism seems to be consistent with the modulatory pathway of escitalopram.

This study also has some limitations. Firstly, the sample size of this study is small, the study population is mild and moderate PSD patients, and the influence of factors such as patients’ education level, economic level and marital status on the prognosis of the disease have not been taken into account, so the effectiveness of electroacupuncture cannot be comprehensively and critically described. Secondly, the inflammatory factors selected in this study were not specific and could be easily interfered with by other inflammatory diseases and drugs. For example, some studies have shown that drugs such as antibiotics (65), NASID (66), and statins (67) can also treat depression through anti-inflammatory mechanisms. However, in this study, there was no difference in the oral medication between the two groups of patients, but it still deserves our concern in future studies. Furthermore, the follow-up period of this study was short and the medium and long-term therapeutic effects of electroacupuncture were not investigated. This suggests that we will need to conduct a large sample, multi-center, medium to long-term follow-up randomized controlled trial using more objective and sensitive study indicators such as functional brain MRI in future studies.

## Conclusion

Electroacupuncture is a safe and effective alternative for patients with mild to moderate PSD and is recommended for clinical use.

## Data availability statement

The raw data supporting the conclusions of this article will be made available by the authors, without undue reservation.

## Ethics statement

The studies involving humans were approved by Ethics Committee of Yancheng Third People’s Hospital on May 28, 2022 (Ethics-Audit-2022-13). The studies were conducted in accordance with the local legislation and institutional requirements. The participants provided their written informed consent to participate in this study. Written informed consent was obtained from the individual(s) for the publication of any potentially identifiable images or data included in this article.

## Author contributions

FM: Methodology, Writing – original draft. CL: Project administration, Writing – review & editing. GC: Data curation, Writing – original draft. LL: Formal Analysis, Writing – original draft. YZ: Funding acquisition, Writing – review & editing. WL: Investigation, Software, Writing – review & editing.

## References

[1] Wang YJ Li ZX Gu HQ Zhai Y Zhou Q Jiang Y. China Stroke Statistics: an update on the 2019 report from the National Center for Healthcare Quality Management in Neurological Diseases, China National Clinical Research Center for Neurological Diseases, the Chinese Stroke Association, National Center for Chronic and Non-communicable Disease Control and Prevention, Chinese Center for Disease Control and Prevention and Institute for Global Neuroscience and Stroke Collaborations. Stroke Vasc Neurol (2022) 7(5):415–50. doi: 10.1136/svn-2021-001374 PMC9614174 35443985

[2] Guo J Wang J Sun W Liu X. The advances of post-stroke depression: 2021 update. J Neurol (2022) 269(3):1236–49. doi: 10.1007/s00415-021-10597-4 34052887

[3] Schöttke H Gerke L Düsing R Möllmann A. Post-stroke depression and functional impairments – A 3-year prospective study. Compr Psychiatry (2020) 99:152171. doi: 10.1016/j.comppsych.2020.152171 32179262

[4] Khedr EM Abdelrahman AA Desoky T Zaki AF Gamea A. Post-stroke depression: frequency, risk factors, and impact on quality of life among 103 stroke patients—hospital-based study. Egyptian J Neurol Psychiatry Neurosurg (2020) 56(1):66. doi: 10.1186/s41983-020-00199-8

[5] Mohammed GF Azab HM Sayed MA-E Elnady HM Youssif H Mahmoud OA-A. Risk factors for post-stroke depression in Sohag University Hospital. Egyptian J Neurol Psychiatry Neurosurg (2019) 55(1):11. doi: 10.1186/s41983-019-0057-z

[6] Jiang X Lin Y Li Y-s. Correlative study on risk factors of depression among acute stroke patients. Eur Rev Med Pharmacol Sci (2014) 18(9):1315–23. 24867509

[7] Maida CD Norrito RL Daidone M Tuttolomondo A Pinto A. Neuroinflammatory mechanisms in ischemic stroke: focus on cardioembolic stroke, background, and therapeutic approaches. Int J Mol Sci (2020) 21(18). doi: 10.3390/ijms21186454 PMC7555650 32899616

[8] Wijeratne T Sales C. Understanding why post-stroke depression may be the norm rather than the exception: the anatomical and neuroinflammatory correlates of post-stroke depression. J Clin Med (2021) 10(8). doi: 10.3390/jcm10081674 PMC8069768 33919670

[9] Kofod J Elfving B Nielsen EH Mors O Köhler-Forsberg O. Depression and inflammation: Correlation between changes in inflammatory markers with antidepressant response and long-term prognosis. Eur Neuropsychopharmacol (2022) 54:116–25. doi: 10.1016/j.euroneuro.2021.09.006 34598835

[10] Feiger JA Snyder RL Walsh MJ Cissne M Cwiek A Al-Momani SI. The role of neuroinflammation in neuropsychiatric disorders following traumatic brain injury: A systematic review. J Head Trauma Rehabil (2022) 37(5):E370–E82. doi: 10.1097/HTR.0000000000000754 35125427

[11] Chen Y Pu J Liu Y Tian L Chen X Gui S. Pro-inflammatory cytokines are associated with the development of post-stroke depression in the acute stage of stroke: A meta-analysis. Top Stroke Rehabil (2020) 27(8):620–9. doi: 10.1080/10749357.2020.1755813 32316861

[12] Chi CH Huang YY Ye SZ Shao MM Jiang MX Yang MY. Interleukin-10 level is associated with post-stroke depression in acute ischaemic stroke patients. J Affect Disord (2021) 293:254–60. doi: 10.1016/j.jad.2021.06.037 34217963

[13] Kim JM Kang HJ Kim JW Bae KY Kim SW Kim JT. Associations of tumor necrosis factor-α and interleukin-1β Levels and polymorphisms with post-stroke depression. Am J Geriatr Psychiatry (2017) 25(12):1300–8. doi: 10.1016/j.jagp.2017.07.012 28844626

[14] Yin J Zhong C Zhu Z Bu X Xu T Guo L. Elevated circulating homocysteine and high-sensitivity C-reactive protein jointly predicts post-stroke depression among Chinese patients with acute ischemic stroke. Clin Chim Acta (2018) 479:132–7. doi: 10.1016/j.cca.2018.01.011 29325799

[15] Rothenburg LS Herrmann N Swardfager W Black SE Tennen G Kiss A. The relationship between inflammatory markers and post stroke cognitive impairment. J Geriatr Psychiatry Neurol (2010) 23(3):199–205. doi: 10.1177/0891988710373598 20601647

[16] Nagy EE Frigy A Szász JA Horváth E. Neuroinflammation and microglia/macrophage phenotype modulate the molecular background of post-stroke depression: A literature review. Exp Ther Med (2020) 20(3):2510–23. doi: 10.3892/etm.2020.8933 PMC7401670 32765743

[17] Lenzi GL Altieri M Maestrini I. Post-stroke depression. Rev Neurol (2008) 164(10):837–40. doi: 10.1016/j.neurol.2008.07.010 18771785

[18] Cai W Mueller C Li YJ Shen WD Stewart R. Post stroke depression and risk of stroke recurrence and mortality: A systematic review and meta-analysis. Ageing Res Rev (2019) 50:102–9. doi: 10.1016/j.arr.2019.01.013 30711712

[19] Yin J Song X Wang C Lin X Miao M. Escitalopram versus other antidepressive agents for major depressive disorder: a systematic review and meta-analysis. BMC Psychiatry (2023) 23(1):876. doi: 10.1186/s12888-023-05382-8 38001423 PMC10675869

[20] Li X Zhang C. Comparative efficacy of nine antidepressants in treating Chinese patients with post-stroke depression: A network meta-analysis. J Affect Disord (2020) 266:540–8. doi: 10.1016/j.jad.2020.02.005 32056924

[21] Cai W Ma W Li YJ Wang GT Yang H Shen WD. Efficacy and safety of electroacupuncture for post-stroke depression: a randomized controlled trial. Acupunct Med (2022) 40(5):434–42. doi: 10.1177/09645284221077104 35232229

[22] Menghan L Bo Z Zhihong M Tao S Yuhui H Hong Z. Effect of Tiaoshen Kaiqiao acupuncture in the treatment of ischemic post-stroke depression: a randomized controlled trial. J Traditional Chin Med (2017) 37(2):171–8. doi: 10.1016/s0254-6272(17)30041-9 29960288

[23] Liu R Zhang K Tong QY Cui GW Ma W Shen WD. Acupuncture for post-stroke depression: a systematic review and meta-analysis. BMC Complement Med Ther (2021) 21(1):109. doi: 10.1186/s12906-021-03277-3 33794857 PMC8017746

[24] Robert GR Jorge RE. Post-stroke depression: A review. Am J Psychiatry (2016) 173(3):221–31. doi: 10.1176/appi.ajp.2015.1503036 26684921

[25] Folstein MF Maiberger R Mchugh PR. Mood disorder as a specific complication of stroke. J Neurol Neurosurg Psychiatry (1977) 40(10):1018–20. doi: 10.1136/jnnp.40.10.1018 PMC492887 591971

[26] Lai Y-J McCullough LD. Chapter 18 - Poststroke Depression: Pathophysiology and Treatment Strategies. In: Quevedo J Carvalho AF Zarate CA, editors. Neurobiology of Depression. Academic Press (2019), p. 197–205.

[27] Loubinoux I Kronenberg G Endres M Schumann-Bard P Freret T Filipkowski RK. Post-stroke depression: mechanisms, translation and therapy. J Cell Mol Med (2012) 16(9):1961–9. doi: 10.1111/j.1582-4934.2012.01555.x PMC3822966 22348642

[28] Yin ZL Ge S Huang LH Cao XX Wu JH. [Acupuncture combined with repetitive transcranial magnetic stimulation for post-stroke depression: a randomized controlled trial]. Zhongguo Zhen Jiu (2022) 42(11):1216–20. doi: 10.13703/j.0255-2930.20211221-0002 36397217

[29] Lu H Li M Zhang B Ren X Meng L Bai W. Efficacy and mechanism of acupuncture for ischemic poststroke depression: Study protocol for a multicenter single-blinded randomized sham-controlled trial. Med (Baltimore) (2019) 98(7):e14479. doi: 10.1097/MD.0000000000014479 PMC6408034 30762770

[30] You Y Zhang T Shu S Qian X Zhou S Yao F. Wrist-ankle acupuncture and Fluoxetine in the treatment of post-stroke depression: a randomized controlled clinical trial. J Tradit Chin Med (2020) 40(3):455–60. doi: 10.19852/j.cnki.jtcm.2020.03.014 32506860

[31] Liu Y Feng H Mo Y Gao J Mao H Song M. Effect of soothing-liver and nourishing-heart acupuncture on early selective serotonin reuptake inhibitor treatment onset for depressive disorder and related indicators of neuroimmunology: a randomized controlled clinical trial. J Tradit Chin Med (2015) 35(5):507–13. doi: 10.1016/s0254-6272(15)30132-1 26591679

[32] Kim JM Stewart R Kim SW Shin IS Kim JT Park MS. Associations of cytokine gene polymorphisms with post-stroke depression. World J Biol Psychiatry (2012) 13(8):579–87. doi: 10.3109/15622975.2011.588247 21793642

[33] Zuo C Cao H Feng F Li G Huang Y Zhu L. Repetitive transcranial magnetic stimulation exerts anti-inflammatory effects *via* modulating glial activation in mice with chronic unpredictable mild stress-induced depression. Int Immunopharmacol (2022) 109:108788. doi: 10.1016/j.intimp.2022.108788 35504201

[34] Yang NN Lin LL Li YJ Li HP Cao Y Tan CX. Potential mechanisms and clinical effectiveness of acupuncture in depression. Curr Neuropharmacol (2022) 20(4):738–50. doi: 10.2174/1570159X19666210609162809 PMC9878952 35168522

[35] Han X Gao Y Yin X Zhang Z Lao L Chen Q. The mechanism of electroacupuncture for depression on basic research: a systematic review. Chin Med (2021) 16(1):10. doi: 10.1186/s13020-020-00421-y 33436036 PMC7805231

[36] Li P Huang W Yan YN Cheng W Liu S Huang Y. Acupuncture can play an antidepressant role by regulating the intestinal microbes and neurotransmitters in a rat model of depression. Med Sci Monit (2021) 27:e929027. doi: 10.12659/MSM.929027 34039946 PMC8168287

[37] Park H Yoo D Kwon S Yoo TW Park HJ Hahm DH. Acupuncture stimulation at HT7 alleviates depression-induced behavioral changes *via* regulation of the serotonin system in the prefrontal cortex of maternally-separated rat pups. J Physiol Sci (2012) 62(4):351–7. doi: 10.1007/s12576-012-0211-1 PMC10717640 22627707

[38] Sun PY Cai RL Li PF Zhu Y Wang T Wu J. [Protective effects on hippocampal neurons and the influence on hippocampal monoamine neurotransmitters with acupuncture for promoting the circulation of the governor vessel and regulating the mental state in rats with post-stroke depression]. Zhongguo Zhen Jiu (2019) 39(7):741–7. doi: 10.13703/j.0255-2930.2019.07.017 31286737

[39] Ning B Wang Z Wu Q Deng Q Yang Q Gao J. Acupuncture inhibits autophagy and repairs synapses by activating the mTOR pathway in Parkinson's disease depression model rats. Brain Res (2023) 1808:148320. doi: 10.1016/j.brainres.2023.148320 36914042

[40] Tu CH MacDonald I Chen YH. The effects of acupuncture on glutamatergic neurotransmission in depression, anxiety, schizophrenia, and alzheimer's disease: A review of the literature. Front Psychiatry (2019) 10:14. doi: 10.3389/fpsyt.2019.00014 30809158 PMC6379324

[41] Le JJ Yi T Qi L Li J Shao L Dong JC. Electroacupuncture regulate hypothalamic-pituitary-adrenal axis and enhance hippocampal serotonin system in a rat model of depression. Neurosci Lett (2016) 615:66–71. doi: 10.1016/j.neulet.2016.01.004 26773866

[42] Miao C Li X Zhang Y. Effect of acupuncture on BDNF signaling pathways in several nervous system diseases. Front Neurol (2023) 14:1248348. doi: 10.3389/fneur.2023.1248348 37780709 PMC10536971

[43] Davila-Hernandez A Gonzalez-Gonzalez R Guzman-Velazquez S Hernandez Hernandez OT Zamudio SR Martinez-Mota L. Antidepressant-like effects of acupuncture *via* modulation of corticosterone, sex hormones, and hippocampal BDNF expression in male rats. Brain Res Bull (2021) 173:53–65. doi: 10.1016/j.brainresbull.2021.05.007 33991609

[44] Cheng WJ Li P Huang WY Huang Y Chen WJ Chen YP. Acupuncture relieves stress-induced depressive behavior by reducing oxidative stress and neuroapoptosis in rats. Front Behav Neurosci (2021) 15:783056. doi: 10.3389/fnbeh.2021.783056 35058758 PMC8763975

[45] Jiang H Deng S Zhang J Chen J Li B Zhu W. Acupuncture treatment for post-stroke depression: Intestinal microbiota and its role. Front Neurosci (2023) 17:1146946. doi: 10.3389/fnins.2023.1146946 37025378 PMC10070763

[46] Chen H Wu C Lv Q Li M Ren L. Targeting mitochondrial homeostasis: the role of acupuncture in depression treatment. Neuropsychiatr Dis Treat (2023) 19:1741–53. doi: 10.2147/NDT.S421540 PMC10404048 37546517

[47] Song C Halbreich U Han C Leonard BE Luo H. Imbalance between pro- and anti-inflammatory cytokines, and between Th1 and Th2 cytokines in depressed patients: the effect of electroacupuncture or fluoxetine treatment. Pharmacopsychiatry (2009) 42(5):182–8. doi: 10.1055/s-0029-1202263 19724980

[48] Zhao YN Zhang S Chen Y Wang Y Chen H Duan YT. Does acupuncture therapy affect peripheral inflammatory cytokines of major depressive disorder? A protocol for the systematic review and meta-analysis. Front Neurol (2022) 13:967965. doi: 10.3389/fneur.2022.967965 36438965 PMC9685430

[49] Li XY Wang HM Zhao Y Li C Lu J Wu JH. [Effect of acupuncture on microglia activation in prefrontal cortex of chronic stress-induced depression rats]. Zhen Ci Yan Jiu (2021) 46(1):52–7. doi: 10.13702/j.1000-0607.200886 33559426

[50] Li X Wang H Li C Wu J Lu J Guo JY. Acupuncture inhibits NLRP3 inflammasome activation in the prefrontal cortex of a chronic stress rat model of depression. Anat Rec (Hoboken) (2021) 304(11):2470–9. doi: 10.1002/ar.24778 34636496

[51] Dong H Qin YQ Sun YC Yao HJ Cheng XK Yu Y. Electroacupuncture Ameliorates Depressive-Like Behaviors in Poststroke Rats *via* Activating the tPA/BDNF/TrkB Pathway. Neuropsychiatr Dis Treat (2021) 17:1057–67. doi: 10.2147/NDT.S298540 PMC8053498 33880028

[52] Halaris A Myint AM Savant V Meresh E Lim E Guillemin G. Does escitalopram reduce neurotoxicity in major depression? J Psychiatr Res (2015) 66-67:118–26. doi: 10.1016/j.jpsychires.2015.04.026 26009299

[53] Avitsur R Paley S Franko M Wolff N Eyal N Doron R. Escitalopram or novel herbal treatments differentially alter cytokine and behavioral responses to immune challenge. J Neuroimmunol (2017) 309:111–8. doi: 10.1016/j.jneuroim.2017.05.020 28601279

[54] Xiaoling Z Yunping H Yingdong L. Analysis of curative effect of fluoxetine and escitalopram in the depression treatment based on clinical observation. Pak J Pharm Sci (2018) 31(3(Special)):1115–8. 29737292

[55] Onal HT Yetkin D Ayaz F. Escitalopram's inflammatory effect on the mammalian macrophages and its intracellular mechanism of action. Prog Neuropsychopharmacol Biol Psychiatry (2023) 125:110762. doi: 10.1016/j.pnpbp.2023.110762 37031947

[56] Sarubin N Nothdurfter C Schmotz C Wimmer AM Trummer J Lieb M. Impact on cortisol and antidepressant efficacy of quetiapine and escitalopram in depression. Psychoneuroendocrinology (2014) 39:141–51. doi: 10.1016/j.psyneuen.2013.10.008 24275013

[57] Knorr U Vinberg M Gether U Winkel P Gluud C Wetterslev J. The effect of escitalopram versus placebo on perceived stress and salivary cortisol in healthy first-degree relatives of patients with depression-A randomised trial. Psychiatry Res (2012) 200(2-3):354–60. doi: 10.1016/j.psychres.2012.05.015 22703642

[58] Benatti C Alboni S Blom JMC Mendlewicz J Tascedda F Brunello N. Molecular changes associated with escitalopram response in a stress-based model of depression. Psychoneuroendocrinology (2018) 87:74–82. doi: 10.1016/j.psyneuen.2017.10.011 29049934

[59] Farahbakhsh Z Radahmadi M. The protective effects of escitalopram on synaptic plasticity in the CA1 region of chronically stressed and non-stressed male rats. Int J Dev Neurosci (2022) 82(8):748–58. doi: 10.1002/jdn.10224 35971746

[60] Chen L Jiang H Bao T Wang Y Meng H Sun Y. Acupuncture ameliorates depressive behaviors by modulating the expression of hippocampal iba-1 and HMGB1 in rats exposed to chronic restraint stress. Front Psychiatry (2022) 13:903004. doi: 10.3389/fpsyt.2022.903004 35733802 PMC9207245

[61] Gong H Su WJ Cao ZY Lian YJ Peng W Liu YZ. Hippocampal Mrp8/14 signaling plays a critical role in the manifestation of depressive-like behaviors in mice. J Neuroinflamm (2018) 15(1):252. doi: 10.1186/s12974-018-1296-0 PMC6122683 30180864

[62] Yue N Li B Yang L Han QQ Huang HJ Wang YL. Electro-acupuncture alleviates chronic unpredictable stress-induced depressive- and anxiety-like behavior and hippocampal neuroinflammation in rat model of depression. Front Mol Neurosci (2018) 11:149. doi: 10.3389/fnmol.2018.00149 29946236 PMC6007169

[63] Stoll G Jander S Schroeter M. Inflammation and glial responses in ischemic brain lesions. Prog Neurobiol (1998) 56(2):149–71. doi: 10.1016/S0301-0082(98)00034-3 9760699

[64] Chen Y Hao C Chen W Cheng W Li P Shen J. Anti-depressant effects of acupuncture: The insights from NLRP3 mediated pyroptosis and inflammation. Neurosci Lett (2022) 785. doi: 10.1016/j.neulet.2022.136787 35820551

[65] Mello BSF Chaves Filho AJM Custodio CS Rodrigues PA Carletti JV Vasconcelos SMM. Doxycycline at subantimicrobial dose combined with escitalopram reverses depressive-like behavior and neuroinflammatory hippocampal alterations in the lipopolysaccharide model of depression. J Affect Disord (2021) 292:733–45. doi: 10.1016/j.jad.2021.05.083 34161892

[66] Yuan Z Chen Z Xue M Zhang J Leng L. Application of antidepressants in depression: A systematic review and meta-analysis. J Clin Neurosci (2020) 80:169–81. doi: 10.1016/j.jocn.2020.08.013 33099342

[67] Dogaru IA Puiu MG Manea M Dionisie V. Current perspectives on pharmacological and non-pharmacological interventions for the inflammatory mechanism of unipolar depression. Brain Sci (2022) 12(10). doi: 10.3390/brainsci12101403 PMC9599138 36291336

